# Drugs and Drink

**Published:** 1895-09-28

**Authors:** 


### Drugs and Drink.

The result of the investigations of the Opium Commission do not promise to be altogether satisfactory to the earnest-minded persons whose reforming zeal has lead to the inquiry. Opium is in the East what alcohol is in the West, and after this we shall have no reason to protest if a band of Mohammedan and Buddhist missionaries come to deal faithfully with us and our spirituous indulgence. The consumption of opium in India is like the consumption of alcohol in Europe—a necessary stimulant to some, a harmless indulgence for many, a fatal vice in a few. Recent returns show that in the asylums of Hindustan the victims of opium-eating are as three to sixteen to those of indulgence in alcohol, and as one to five to those of bhang.

It seems then that opium, whatever its dangers, is less harmful to Asiatics than alcohol. Our pity and terror, founded on Western experience, or perhaps Western inexperience, are wasted, and our Indian fellow-subjects are in no greater danger than ourselves of degradation, crime, and insanity, due to indulgence in liquor. As a matter of fact, an imported indulgenee is usually more dangerous than a native one; what one climate and temperament will stand another will not, and we might do most for health, wealth, and wisdom by sending our enthusiastic teetotallers to India, and establishing opium commissions at home. Statistics have shown that the greatest drinkers among the nations are not by any means the most degraded and effete, and it is matter of common notoriety that wine-producing countries are not the abodes of drunkenness. Scotch shepherds and gamekeepers consume an amount of whisky that frightens shooting tenants, who do not realise that an open-air life among the hills and moorlands gives a different constitution from that of London, yet it is not from "the lone shieling in the misty island" that the tale comes of mad intoxication, unthrift, cruelty, and crime; but from the slum, where some liquor, concocted by the chemist rather than the distiller, acts like a witches' brew on a physique already poor and shattered.

Stimulants are the natural indulgence of the strong and stolid, narcotics of the weak and sensitive. The Viking, giver and receiver of hard blows, thick-skinned in body and mind, fired his imagination with mead and saw the violent glories of Valhalla. The Hindu sage, weak with fasting, steadied his shaking nerves with opium, and then, in "dreams that wave before the half-shut eye" beheld Nirvana, the heavenly nothingness. The dullard required something to rouse his imagination, the over-sensitive dreamer something to soothe his nerves, and thus drunkenness is a vice of savagery, drugging a vice of civilisation.

With our civilisation has come a great and continual decrease in drunkenness. The "national drink bill," concerning which so much is said, is less, man for man, than it was a century or half a century ago, or at least represents a less consumption. We hear more about drunkenness, but that is because we have ceased to regard it as a matter of course, a regular part of daily life. Therefore, it may be surmised that our blue-ribbon and other armies in fighting the "drink-fiend" are striking at what is, after all, a dying monster, though dangerous even in the death-grips. Meanwhile another and subtler demon arises. The vices of civilisation are with us, and those who would scorn to "drink," as they call it—differentiating the consumption of alcohol from that of all other liquids—indulge in drugs of various kinds. Opium, either in the form of laudanum-drinking or in the subtler form of the hypodermic injection of morphia, is, though not common, less rare than it is guessed to be. Insomnia or pain which to our sensitive nerves seems intolerable, is the excuse for beginning the habit, and the end is worse than drunkenness. The "cocaine habit" is a recognised fact in America, where our Teutonic race, subjected to an intenser climate and an intenser life than in Europe, has developed a quicker sensibility and more irritable nerves. The inhalation of chloroform has proved an irresistible temptation to many, and women who would scorn any indulgence in wine sip eau de Cologne and other perfumes. Infinitely more dangerous than alcohol are these forms of inebriety in that no revolting associations surround them. Their victims do not regard themselves as drunkards—at least, not until the habit is fully formed. They cannot identify their nervousness, their faintness, their sleeplessness with the cravings of the staggering loafer of the street, nor the rest they obtain after the dose with the stupor of vulgar drunkenness. Therefore, to an extent which is not generally realised, women are subject to this temptation. Their imagination is not offended, and they can usually obtain the drug they want without suspicion, and carry it about in convenient forms. It takes a long time for even intimates to realise that the depression, the irritability, the varying moods of a rigid abstainer arise from drunkenness; we generally conceive that condition as being due to only one source. And drug habits of various sorts become incurable just because no one realises that they are simply a form of inebriety.

The general habit of drugging is to be condemned. No healthy person needs continual medicating with either digestives, purgatives, tonics, or sedatives. If a doctor prescribes these things, good and well; he knows, presumably, when to give and when to stop, but the irresponsible way in which people pour substances far stronger than alcohol into their systems would awaken one's admiration for their courage, if it did not arouse one's indignation at their folly. Cases have been known of ginger drunkenness. Extracts of ginger, popular among women as relieving functional disturbances, contain strong alcohol disguised by the pungent spice. Arsenic-eating is not confined to Styria, but is popular as an improvement to the complexion among the society dames of Australia, while innocent people take strychnine in their tonics, without knowing it, until they begin to feel "jumpy." The danger is twofold—first, the directly injurious effects of the chosen drug, and secondly the risk of ignorantly clasping to one's breast a viper more malignant than the "drink fiend." Alcohol we know and dread, but these things—as dangerous as alcohol—we take without any fear. And while for men and women of a different race, dwelling in a hot and malarious climate, quinine, opium, and other drugs may be not only harmless but even wholesome, they are for us Anglo-Saxons, except in rare instances, subtle and pernicious foes.

